# Quantifying side effects and caregiver burdens of pediatric pulmonary hypertension therapies

**DOI:** 10.1186/s12887-023-03860-2

**Published:** 2023-01-25

**Authors:** Erik J. Nelson, Ella Cook, Samara Nelson, Rebecca Brown, Megan Pierce, Ashley Bangerter Seelos, Heather Stickle, Michael Johansen

**Affiliations:** 1 Department of Public Health, Brigham Young University, 2148 LSB, Provo, UT 84660 USA; 2 Emma Eccles Jones College of Education & Human Services, Utah State University, Logan, UT USA; 3 Indiana University School of Medicine, Indianapolis, IN USA

**Keywords:** Pulmonary hypertension, Treatment side effects, Facebook, Access to care, Caregiver burden

## Abstract

**Background and objectives:**

Pulmonary hypertension (PH) is a rare, but serious disease among children. However, PH has been primarily evaluated among adults. Consequently, treatment therapies have not been fully evaluated among pediatric populations and are used in an ‘off label’ manner. The purpose of this study was to estimate the side effect profiles of the most commonly prescribed pediatric PH therapies and to understand the burdens placed upon families caring for children living with PH.

**Methods:**

Participants were recruited online through the “Families of children with pulmonary hypertension” Facebook group and asked to complete a survey about PH treatments.

**Results:**

A total of 139 parents of a child living with PH completed the survey. Almost all children used ≥ 1 medication to treat PH, with 52% using ≥ 3 medications. The highest average number of side effects was reported by users of Treprostinil, Selexipag and type-5 phosphodiesterase (PDE_5_) inhibitors. The most common side effects were skin flushing, headache, nasal congestion, joint/muscle pain, and nausea. In terms of accessing care, 81% travel ≥ 20 miles and 68% travel for ≥ 60 min to receive care.

**Conclusions:**

We found an array of treatment combinations employed to mitigate symptoms of PH in children, with a wide range of side effects. We also found a large, unseen economic, emotional, and time burden of caring for a child living with PH. Further research is warranted to understand the clinical implications of these side effects to move towards labeled usage of these therapies rather than post-hoc off-label usage.

## Article summary

This study captures the usage and side effects of therapies for children living with pulmonary hypertension.

## What’s known on this subject

Pulmonary hypertension is a rare pediatric condition that is treated with off-label usage of medications that have been approved for use in adults.

## What this study adds

This study highlights side effects of pulmonary hypertension therapies in children, both physiologically and financially.

## Introduction

Pulmonary hypertension (PH) is rare but serious and potentially a life-threatening disease characterized by an elevation in mean pulmonary artery pressure and pulmonary vascular resistance [1]. In severe cases, PH leads to right heart failure, clinical worsening, and death. Despite an estimated annual incidence rate of approximately 3 cases of PH per million children, PH remains an important cause of morbidity and mortality among children [1, 2]. Etiologies are numerous and include chronic infections, lung disease and chronic hypoxemia, thromboembolic disease, and genetic and metabolic abnormalities, but it is most commonly idiopathic or associated with congenital heart disease [3–10]. In children, untreated PH carries a grim prognosis, with subtypes such as idiopathic PH predicting a median survival of just 10 months [11]. PH has been reported in 23 to 37% of premature infants with chronic lung disease in multiple retrospective studies, and carries a 2-year mortality rate as high as 48% [12–15]. In addition to substantial mortality risk, PH associated with chronic lung disease is associated with increased morbidity such as prolonged mechanical ventilation, need for supplemental oxygen, and increased hospital length of stay [12–19].

Despite recent developments in PH-specific therapies, survival of patients with idiopathic PH remains poor and appears to be worse in children compared to adults [11]. PH has primarily been characterized and studied among adults and therefore the development of treatment therapies has also targeted this population [20]. An array of pharmacologic treatments are available and approved for use in adults, but studies evaluating therapeutic dosing, side effects, and outcome for pediatric patients are lacking. PH clinicians are nonetheless prescribing these medications off-label in children as part of standard practice, often with guidance from only small cohort studies or past clinical experience [21]. However, benefit is frequently seen [22]. Combination drug therapy is increasing in frequency, again, backed by large adult studies [20, 23]. Many factors such as disease severity, side effect profile, drug interaction, cost, and impact on quality of life must be considered when prescribing for the pediatric population. Three classes of drugs have been extensively evaluated for the pharmacologic treatment of pediatric PH: prostanoids (epoprostenol, treprostinil, iloprost, beraprost), endothelin receptor antagonists (ERAs) (bosentan, ambrisentan), and type-5 phosphodiesterase (PDE_5_) inhibitors (sildenafil, tadalafil). Because vasoconstriction is an important component in the development of PH, vasodilator drugs are also frequently used to decrease pulmonary arterial pressure, to improve cardiac output, and to potentially reverse pulmonary vascular changes in the lungs [23–28]. However, the adverse effects of these drugs have often been studied in isolation and in clinical trial settings [23, 27, 28]. With increased usage of novel PH treatment therapies (such as Treprostinil and Selexipag), there is a urgent need to better understand the adverse effects experienced by pediatric patients in every day settings where complex combined therapy regimens are commonplace [21].

In addition to the physiological burdens and side effects of PH treatment therapies, there are social, economic, emotional and health access implications for children living with PH, their families, and their caregivers. Children who live in remote settings have limited access to emergency and specialty care [29]. Accessing care is even more limited for children living with PH as specialty PH care centers and physicians with specialized PH training are much more sparse than general clinics and hospitals [30]. This is of particular interest as PH treatment therapies side effects may greatly affect a family’s ability to thrive while caring for a child living with PH [31].

The purpose of our study was to estimate the side effect profiles of the most commonly prescribed PH therapies among children and to understand the burdens placed upon families to access care for children living with PH.

## Methods

### Facebook recruitment campaign

Participants were recruited online via invitations posted on the “Families of children with pulmonary hypertension” Facebook™ page. To maximize study participation and to provide each group member an opportunity to participate, we posted the invitation on the Facebook group homepage 3 times over a 5-week period during December 2021 to January 2022. A random sample of group members (934 out of 1593 total members) also received a personal invitation via the internal Facebook messaging system (Facebook Messenger). Participants who clicked on the invitation link were provided with study information, asked to convey consent to participate, and then immediately proceeded to begin the survey using the Qualtrics™ survey platform. Eligible individuals who completed the survey were compensated for their participation and emailed a $20 gift card to Amazon.com®.

### Study participants

Parents and caregivers of children living with PH were surveyed about their personal experiences, as well as those of their child living with PH. Participants were English-speaking, aged 18 years or older, members of the “Families of children with pulmonary hypertension” Facebook group, and self-identified as parents, guardians or primary caregivers of a child living with PH. This Facebook group was selected because it is the largest known gathering place for parents of children living with PH (with 1593 total group members), many of whom routinely (daily) interact with others to learn more about existing PH therapies, to cope, and to share experiences of caring for a child with PH.

### Survey instrument

We developed a questionnaire containing questions intended to be answered by the parent of a child living with PH. The survey assessed age at PH diagnosis, congenital heart defects, presence of Down syndrome, maternal age at birth, gestational age, PH therapies utilized, and side effects attributed to PH medications (Treprostinil, Tadalafil, Sildenafil, Ambrisentan, Bosentan, Macitentan, Selexipag, and Riociguat) used during the past 6 months. We also queried participants’ knowledge, attitudes, and perceptions of the Potts Shunt procedure and combined heart/lung transplant. We also assessed specific access to care issues and care provider characteristics such as where PH care is received (local pediatrician’s office, specialty children’s hospital, no specialty PH care available), the type of PH care provider (pediatric cardiologist, pediatric pulmonologist, PH specialist, combination of cardiologist and pulmonologist), distance traveled (miles) to primary PH provider, travel time to PH provider (minutes), need to move residence to receive PH care for child (yes/no), whether genetic testing was offered at diagnosis (yes/no), whether genetic testing was performed (yes/no) and whether a genetic counselor discussed any genetic testing results (yes/no). Finally, we queried family sociodemographics including race (white/other), relationship to child living with PH (father, mother, guardian, primary caregiver), U.S. citizenship (yes/no), respondents’ educational attainment (high school, some college, associate’s degree, bachelor’s degree, graduate or professional degree), employment status (employed, homemaker, unemployed), household income (< $35,000, $35,000–74,999, > $75,000), and health insurance status (yes/no). The average time to complete the survey was 19.5 min (range: 8 to 56 min).

### Statistical analysis

We report the counts and frequencies (percentages) of all medications used by children living with PH. We also report the mean (standard deviation) number of medications used, as well as the side effects reported by parents/caregivers of children living with PH associated with each medication. Statistical analysis was conducted in R version 4.1.0 [32].

## Results

A total of 139 adults who identified as the parent or primary caregiver of a child living with PH responded to the online survey. Respondents self-reported that they were the child’s mother (89.2%), white (82.0%), had achieved a college degree or higher (60.5%), were residents of the United States (85.6%), and employed (64.0%) or a homemaker (24.5%) (see Table 1). As for the children with PH, the majority were ≥ 36 weeks of gestation at birth (62.6%), diagnosed before one year of age (49.6%), born with a congenital heart defect (48.2%, see Table 2 for complete listing of defects), and 8.6% of children living with PH also being diagnosed with Down syndrome (Table 3). Upon diagnosis of PH, 81 children (58.3%) living with PH were offered genetic testing, of which 76 (93.8%) elected to have genetic testing performed and only 63 (82.9%) had a genetic counselor with whom to discuss the test results.

**Table 1:** Characteristics of parents/caregivers of children living with pulmonary hypertension (*n* = 139)

Survey Respondent Characteristics	n (%)
**Survey respondent’s age (years)**	
Mean (SD)	39.4 (8.73)
**Child’s sex**	
Female	63 (45.3%)
Male	76 (54.7%)
**Relationship to child with PH**	
Father	10 (7.2%)
Guardian	3 (2.2%)
Mother	124 (89.2%)
Primary caregiver	2 (1.4%)
**Respondent's educational attainment**	
High school graduate (includes equivalency, i.e., GED)	9 (6.5%)
Some college, no degree	26 (18.7%)
Associate’s degree	18 (12.9%)
Bachelor’s degree	45 (32.4%)
Graduate or professional degree	39 (28.1%)
Prefer not to say	2 (1.4%)
**Employment status**	
Employed	89 (64.0%)
Homemaker	34 (24.5%)
Unemployed	14 (10.1%)
Prefer not to say	2 (1.4%)
**Household income**	
< $35,000	16 (11.5%)
$35,000 to $74,999	37 (26.6%)
> $75,000	76 (54.7%)
Prefer not to say	10 (7.2%)
**Home ownership**	
Own (or paying mortgage)	108 (77.7%)
Rent	30 (21.6%)
Prefer not to say	1 (0.7%)
**How long lived in current residence**	
Less than 1 year	11 (7.9%)
1 to 2 years	16 (11.5%)
2 to 3 years	22 (15.8%)
3 to 5 years	20 (14.4%)
5 or more years	69 (49.6%)
Prefer not to say	1 (0.7%)

**Table 2:** Birth defects reported by parents/caregivers of children living with pulmonary hypertension (*n* = 67)

	**N (%)**
**Parent/Guardian Reported Birth Defect**	
Yes	67 (48.2)
No	72 (51.8%)
**Total # of Birth Defects Reported**	119
**# of Birth Defects Reported per Child (mean, SD)**	1.13 (1.58)
**Birth defects reported by respondents**^a^	
***Left Heart Obstructive Lesions***	
Coarctation of the Aorta	4 (6%)
Aortic Valvar Stenosis (AS)	1 (1.5%)
Interrupted Aortic Arch / VSD	2 (3%)
Shone's Complex	2 (3%)
***Conotruncal Abnormalities***	
Pulmonary Atresia (PA)	1 (1.5%)
Pulmonary Valvar Stenosis (PS)	1 (1.5%)
Taussig-Bing Variant DORV	1 (1.5%)
Tetralogy of Fallot	3 (4.5%)
Transposition of the Great Arteries	10 (14.9%)
Absent left pulmonary artery	1 (1.5%)
***Pulmonary Venous Abnormalities***	
Scimitar syndrome	1 (1.5%)
Partial Anomalous Pulmonary Venous Return (PAPVR)	2 (3%)
Total Anomalous Pulmonary Venous Return (TAPVR)	1 (1.5%)
***Systemic Venous Abnormalities***	
Persistent left superior vena cava	1 (1.5%)
***Septation Defects and Extracardiac Shunts***	
Atrial Septal Defect (ASD)	28 (41.8%)
Atrioventricular Septal Defect (or AV Canal Defect)	8 (11.9%)
Patent Ductus Arteriosus (PDA)	21 (31.3%)
Ventricular Septal Defect (VSD)	20 (29.9%)
Patent Foramen Ovale (PFO)	3 (4.5%)
***Single Ventricle Physiology***	
Hypoplastic Left Heart Syndrome	2 (3%)
Single Ventricle Anomalies NOS	2 (3%)
***Airway/Respiratory Abnormalities/Other***	
Bronchopulmonary Dysplasia (BPD)	2 (3%)
Bronchomalacia	1 (1.5%)
Laryngomalacia	1 (1.5%)

^a^Percentages are calculated among the 67 children with birth defect(s)

**Table 3:** Characteristics of children living with pulmonary hypertension in the study sample (*n* = 139)

Child's Characteristics	n (%)
**Child’s race/ethnicity**	
White/Caucasian	114 (82.0%)
Other	25 (18.0%)
**Hispanic**	
Yes	6 (4.3%)
No	133 (95.7%)
**Citizenship status**	
U.S. Resident	119 (85.6%)
Outside U.S	15 (10.8%)
Prefer not to say	5 (3.6%)
**Gestational age at birth**	
Less than 24 weeks	6 (4.3%)
More than 36 weeks	87 (62.6%)
24 to 28 weeks	12 (8.6%)
29 to 32 weeks	4 (2.9%)
33 to 36 weeks	21 (15.1%)
I don’t know	9 (6.5%)
**Age at PH diagnosis**	
First 30 days	32 (23.0%)
1–12 months	37 (26.6%)
1–4 years	40 (28.8%)
5–10 years	23 (16.5%)
11–15 years	7 (5.0%)
**Current age**	
< 1 year	13 (9.4%)
1–5 years	45 (32.4%)
6–10 years	36 (25.9%)
11–15 years	28 (20.1%)
16–17 years	15 (10.8%)
> = 18 years	2 (1.4%)
**Down Syndrome**	
Yes	12 (8.6%)
No	126 (90.6%)
Prefer not to say	1 (0.7%)
**Congenital heart defect**	
Yes	67 (48.2%)
No	72 (51.8%)
**Health insurance**	
Yes	131 (94.2%)
No	6 (4.3%)
Prefer not to say	2 (1.4%)
**Offered genetic testing at diagnosis**	
Yes	81 (58.3%)
No	54 (38.8%)
I don’t know	3 (2.2%)
Prefer not to say	1 (0.7%)
**Genetic testing performed**	
Yes	76 (93.8%)
No	5 (6.2%)
**Genetic counselor discussed results**	
Yes	63 (82.9%)
No	9 (11.8%)
I don’t know	3 (3.9%)
Prefer not to say	1 (1.3%)

The majority (89.9%) of children living with PH used at least one medication to treat PH, with 51.8% using ≥ 3 medications (mean = 2.25 medications, SD = 1.18). In addition to pharmaceutical interventions, 60.4% of children living with PH reported using supplemental oxygen (see Table 4). Among the 84 children who used supplemental oxygen, 28.7% used it continuously, 38.3% at nighttime only, and 12.8% used supplemental oxygen as needed. The highest average number of side effects was reported by treprostinil (Remodulin™) users (4.40), selexipag (Uptravi™) users (2.85) and type-5 phosphodiesterase (PDE_5_) inhibitor users (tadalafil/Adcirca™ = 2.40, sildenafil/Revatio™ = 1.61). Endothelin receptor antagonist (ERAs) medications had the lowest average number of side effects reported (ambrisentan/Letairis™ = 0.53, bosentan/Tracleer™ = 0.26 and macitentan/Opsumit™ = 0.67). Across all medications, the most commonly reported side effects during the past 6 months were flushing of the skin (*n* = 106, 18.6%), headache/dizziness (*n* = 65, 11.4%), nasal congestion (*n* = 47, 8.3%), joint/muscle pain (*n* = 46, 8.1%), and nausea (*n* = 45, 7.9%). PDE_5_ medications were associated with the greatest proportion of side effects for flushing of the skin (*n* = 61, 51.2%), headache/dizziness (*n* = 34, 28.6%), nasal congestion (*n* = 47, 39.5%), nausea (*n* = 32, 26.9%), heartburn (*n* = 32, 26.9%), difficulty sleeping (*n* = 24, 20.2%) and nose bleeds (*n*= 26, 21.8%), which was similar to what has been reported elsewhere [21].

**Table 4:** Medications used by children living with pulmonary hypertension (*n* = 139)

Medication	n (%)
**Number of medications used**	
0	14 (10.1%)
1	25 (18.0%)
2	28 (20.1%)
3	56 (40.3%)
4	16 (11.5%)
Mean (SD)	2.25 (1.18)
**Oxygen usage**	
Yes	84 (60.4%)
No	55 (39.6%)
**When oxygen is used**	
At nighttime only	36 (38.3%)
All the time (continuously)	27 (28.7%)
As needed	12 (12.8%)
When sick	7 (7.4%)
At high altitude	5 (5.3%)
Only when they are under exertion (moving)	4 (4.3%)
Eating	2 (2.1%)
When not in School	1 (1.1%)
**Aspirin**	
Yes	33 (23.7%)
No	105 (75.5%)
I don’t know	1 (0.7%)

Parents/caregivers reported that children living with PH primarily receive routine care at a specialty children’s hospital (95.7%), and from disease specialists, including pediatric cardiologists (40.3%), pediatric pulmonologists (7.2%), a combination of pediatric cardiologists and pulmonologists in a joint office visit (43.9%), or from a general PH specialist (7.2%). In terms of accessing care, 81.3% travel ≥ 20 miles to receive care, 68.3% travel for ≥ 60 min to receive routine care, and 8.6% of families relocated their home in order to be closer to routine PH care.

## Discussion

The objective of this study was to estimate the side effect profiles of the most commonly prescribed PH therapies among children and to better understand the burden on families to access care for children living with PH. Our findings indicate that several treatment combinations are employed to mitigate symptoms of PH in children, resulting in a wide range of side effects experienced by children. Over half of children living with PH were using at least 3 medications to manage their PH, thus the need for understanding treatment side effects in this population is significant. We used previously published data [33–36] to compare reported side effects among medication classes. When comparing treprostinil (Remodulin) use in children to adults, we found children to report more side effects than adults for infusion site pain (95.3% vs. 85%), diarrhea (34.9% vs. 25%), rash (37.2% vs. 14%), flushing of the skin (72.1% vs. 11%) and swelling/edema (62.7% vs. 9%). For selexipag (Uptravi), the side effects reported by children in our study were lower than those reported among adults for headache (46.1% vs. 65%), diarrhea (38.5% vs. 42%), and similar for nausea (38.5% vs. 33%), yet much higher for joint/muscle pain (46.1% vs. 17%) and flushing of the skin (50.0% vs. 12%) (see Table 5). Importantly, these side effect comparisons juxtapose combination therapy use in our study to studies conducted in clinical settings, which may partially explain the differences observed as combination therapies have been associated with increased side effects [23, 24, 26]. These reported differences in side effects among children and adults living with PH highlight important considerations for physicians as they prescribe these medications in an attempt to balance the management of disease symptoms and quality of life experiences.

**Table 5:** Number of pulmonary hypertension medicinal therapies used and their side effects as reported by parents/caregivers of children living with pulmonary hypertension (*n* = 139)

	Parenteral Prostacyclin	Phosphodiesterase Inhibitors (PDE 5)		Endothelin Receptor Antagonists (ERAs)			Oral Prostacyclin Receptor Agonist	Inhaled Prostacyclin	Soluble Guanylate Cyclase
	Treprostinil (Remodulin)	Tadalafil (Adcirca)	Sildenafil (Revatio)	Ambrisentan (Letairis)	Bosentan (Tracleer)	Macitentan (Opsumit)	Selexipag (Uptravi)	Treprostinil (Tyvaso)	Riociguat (Adempas)
**Number of children taking Medication**	43	70	49	56	19	12	26	2	2
**Total # of Side Effects Reported**	190	183	74	30	5	8	74	2	3
**Average # of Side Effects per Child (mean, SD)**	4.40 (2.44)	2.40 (1.20)	1.61 (0.86)	0.53 (0.90)	0.26 (0.56)	0.67 (1.50)	2.85 (2.13)	1.0 (0.0)	1.50 (0.71)
**Parent/Guardian Reported ≥ 1 Side Effect**									
Yes	42 (97.7%)	62 (88.6%)	36 (73.5%)	19 (33.9%)	4 (21.1%)	3 (25.0%)	21 (80.8%)	1 (50.0%)	1 (50.0%)
No	1 (2.3%)	8 (11.4%)	13 (26.5%)	37 (66.1%)	15 (78.9%)	9 (75.0%)	5 (19.2%)	1 (50.0%)	1 (50.0%)
**Side Effects Reported**									
Flushing of the skin	31 (72.1%)	35 (50.0%)	26 (53.1%)	-	-	-	13 (50.0%)	1 (50.0%)	-
Difficulty sleeping	2 (4.7%)	16 (22.9%)	8 (16.3%)	-	-	-	-	-	-
Headache/Dizziness	11 (25.6%)	28 (40.0%)	6 (12.2%)	4 (7.1%)	1 (5.3%)	3 (25.0%)	12 (46.1%)	-	-
Low blood pressure	1 (2.3%)	-	1 (2.0%)	-	-	-	1 (3.8%)	-	-
Nasal congestion	-	37 (52.8%)	10 (20.4%)	-	-	-	-	-	-
Nausea	-	24 (34.3%)	8 (16.3%)	-	-	-	12 (46.1%)	-	1 (50.0%)
Diarrhea	15 (34.9%)	-	-	-	-	-	10 (38.5%)	-	1 (50.0%)
Vomiting	1 (2.3%)	-	-	-	-	-	3 (11.5%)	-	-
Heartburn	-	24 (34.3%)	8 (16.3%)	-	-	-	-	-	1 (50.0%)
Nose bleeds	-	19 (27.1%)	7 (14.3%)	-	-	-	-	-	-
Chest pain	-	-	-	7 (12.5%)	-	-	-	-	-
Fever	4 (9.3%)	-	-	1 (1.8%)	-	-	-	-	-
Irregular heart beat	-	-	-	1 (1.8%)	1 (5.3%)	-	-	-	-
Itching	19 (44.2%)	-	-	3 (5.4%)	-	1 (8.3%)	-	-	-
Joint/Muscle Pain	20 (46.5%)	-	-	10 (17.9%)	2 (10.5%)	2 (16.6%)	12 (46.1%)	-	-
Sore throat	-	-	-	1 (1.8%)	-	1 (8.3%)	-	1 (50.0%)	-
Swelling	27 (62.7%)	-	-	3 (5.4%)	1 (5.3%)	-	-	-	-
Fainting	-	-	-	-	-	1 (8.3%)	-	-	-
Fatigue	-	-	-	-	-	-	1 (3.8%)	-	-
Jaw pain	-	-	-	-	-	-	3 (11.5%)	-	-
Decreased Appetite	1 (2.3%)	-	-	-	-	-	5 (19.2%)	-	-
Low red blood cell count	-	-	-	-	-	-	2 (7.7%)	-	-
Rash	16 (37.2%)	-	-	-	-	-	-	-	-
Scars	1 (2.3%)	-	-	-	-	-	-	-	-
Tenderness around infusion site	41 (95.3%)	-	-	-	-	-	-	-	-

Another important finding of this study is that PDE_5_ medications such as tadalafil (Adcirca) and sildenafil (Revatio) were associated with higher frequencies of side effects for children living with PH than those listed among adult populations in the package insert. For example, 50–53% of children in our study reported flushing of the skin, compared to 2–3% (tadalafil) and 4% (sildenafil) in the package inserts. Similarly, the two most commonly reported side effects in the package insert for tadalafil were headaches (11–15%) and nasal congestion (1–3%), yet 40.0% of children in our study reported experiencing headaches and 52.8% reported nasal congestion. The results were similar comparing children in our study to the sildenafil package insert, with headache (12.2% vs. 4%), nasal congestion (20.4% vs. 9%) and flushing of the skin (53.1% vs. 4%) all being considerably higher than among adults. This finding is important as PDE_5_ medications are frequently first-line therapies and considered to have a low side effect profile. Our findings suggest that they may contribute to significant side effects and impact quality of life for children living with PH, even though they are relatively mild compared to the side effects reported by other PH medications.

In addition to the pharmaceutical side effects, this study aimed to understand the unseen and often ignored burden of caring for a child living with PH (see Table 6). Due to the rare occurrence and highly specialized treatment of PH in children, almost all (95.7%) of the children in our study reported receiving routine care at a specialty children’s hospital. However, in order to access this care, 81.3% of families had to travel ≥ 20 miles to receive care, with 68.3% of families travelling for more than 60 min (one-way) to receive care. Chien et al. recently reported that 90.6% of children live within a 60-min drive of general pediatric inpatient services, though only 39.6% lived within a 60-min drive of a pediatric emergency hospital [29]. Further complicating matters, these drive times tend to increase for rural, lower income and minority populations [29, 37, 38]. One of the most stark findings of our study is that 8.6% (*n* = 12) of families had to relocate once their child was diagnosed with PH. Moreover, 71.2% of families report spending more than 30 min ordering specialty medications by phone each month (data not shown). In addition, 8.6% of families reported difficulty covering the treatment costs, despite 94.2% having health insurance coverage, and 26.6% of families had sought financial aid from various charitable organization to assist with co-pay and direct costs of PH treatment. Thus, the need for transportation, the large time commitment required to travel to/from primary care appointments, home location, and financial impacts on families of children with PH cannot go unnoticed.

**Table 6:** Care provider characteristics and access reported by parents/caregivers of children living with pulmonary hypertension (*n* = 139)

**Care Provider Characteristics**	n (%)
**Where PH care is received**	
Local pediatrician’s office	2 (1.4%)
Specialty Children’s Hospital	133 (95.7%)
There is no PH care where we live	4 (2.9%)
**Type of PH provider**	
Pediatric cardiologist & pediatric pulmonologist	61 (43.9%)
Pediatric Cardiologist	56 (40.3%)
Pediatric Pulmonologist	10 (7.2%)
PH Specialist	10 (7.2%)
Primary care physician (Local Pediatrician)	2 (1.4%)
**Distance to primary PH provider**	
Less than 5 miles	4 (2.9%)
6–10 miles	8 (5.8%)
11–20 miles	13 (9.4%)
More than 20 miles	113 (81.3%)
Prefer not to say	1 (0.7%)
**Travel time to PH provider**	
Less than 10 min	3 (2.2%)
10–20 min	5 (3.6%)
21–30 min	11 (7.9%)
30–45 min	15 (10.8%)
46–60 min	9 (6.5%)
61–90 min	21 (15.1%)
More than 90 min	74 (53.2%)
Prefer not to say	1 (0.7%)
**Had to move residence to be closer to PH care provider**	
Yes	12 (8.6%)
No	127 (91.4%)

Limitations exist for our study, as the data used in our analysis were self-reported and may be subject to under- or over-reporting. Participants were recruited via the Internet from a Facebook group, which may have resulted in selection bias. However, this is the largest known gathering for parents/caregivers of children living with PH and was the most direct way at locating a sufficient sample of children with this rare condition. It should also be noted that other Internet-based studies have demonstrated validity and reduction in potential biases such as interviewer bias and social desirability bias [39, 40]. In addition, we were unable to collect dosage for each medication and therefore cannot determine the dose-dependent side effects. Finally, we asked parents/caregivers to report side effects experienced during the past 6 months for each medication their child living with PH used, which may not adequately capture all side effect events that occurred. This reporting also constrained the parents/caregivers to associate experienced side effects with each medication, which means that some reported side effects could have been potentially double counted across medications and/or misclassified by drug type.

## Conclusion

Overall, the findings from this study have important implications for families seeking treatment for pediatric PH and for their physicians. First, PH medications have several known side effects, though the frequency may be different from that experienced by adults. Second, the side effects of each medication should be carefully considered in consultation with a licensed medical provider. Third, significant unseen familial burdens accompany a child’s diagnosis of PH and should not be overlooked. Further research is needed to better understand the clinical implications of these side effects, insurance coverage of these medications, and moving towards labeled usage of these therapies rather than post-hoc off-label usage.

## Data Availability

We are not allowed to post the data to a repository (even de-identified) due to HIPAA concerns (potential to identify subjects). Data and materials available upon request to the corresponding author.

## Abbreviations

PH: Pulmonary hypertension
PDE_5_: Type-5 phosphodiesterase inhibitors
ERA: Endothelin receptor antagonist
